# Immunomodulatory Effect of MSCs and MSCs-Derived Extracellular Vesicles in Systemic Lupus Erythematosus

**DOI:** 10.3389/fimmu.2021.714832

**Published:** 2021-09-16

**Authors:** Chunjuan Yang, Jianmei Sun, Yipeng Tian, Haibo Li, Lili Zhang, Jinghan Yang, Jinghua Wang, Jiaojiao Zhang, Shushan Yan, Donghua Xu

**Affiliations:** ^1^Department of Rheumatology of the First Affiliated Hospital, Weifang Medical University, Weifang, China; ^2^Central Laboratory of the First Affiliated Hospital, Weifang Medical University, Weifang, China; ^3^Department of Chemistry, School of Applied Chemistry, Food and Drug, Weifang Engineering Vocational College, Qingzhou, China; ^4^Material Procurement Office of the First Affiliated Hospital, Weifang Medical University, Weifang, China; ^5^Department of Gastrointestinal and Anal Diseases Surgery of the Affiliated Hospital, Weifang Medical University, Weifang, China

**Keywords:** mesenchymal stem cells, extracellular vesicles, exosome, systemic lupus erythematosus, immunity

## Abstract

Systemic lupus erythematosus (SLE) is a common autoimmune connective tissue disease with unclear etiology and pathogenesis. Mesenchymal stem cell (MSC) and MSC derived extracellular vesicles (EVs) play important roles in regulating innate and adaptive immunity, which are involved in many physiological and pathological processes and contribute to the immune homeostasis in SLE. The effects of MSCs and EVs on SLE have been drawing more and more attention during the past few years. This article reviews the immunomodulatory effects and underlying mechanisms of MSC/MSC-EVs in SLE, which provides novel insight into understanding SLE pathogenesis and guiding the biological therapy.

## Introduction

SLE is a systemic autoimmune disease with multiple organs and multiple systems damages. It is characterized by abnormal activation of immune cells, abundant production of pathogenic autoantibodies and immune complexes deposition (1). The etiology and pathogenesis of SLE are complex and accompanied by immune disorders including abnormal proliferation, differentiation, activation and dysfunction of T cells, mononuclear-macrophage cells as well as B cells. Long term autoimmune disorders and sustained inflammation eventually cause tissue and organ damages (2, 3). Lupus nephritis is the most common and severe organ injury in SLE (4). At present, glucocorticoids and immunosuppressants are traditional treatments for SLE. However, there are still many refractory patients who are difficult to achieve clinical remission with high mortality. It brings a great economic burden and psychological pressure to SLE patients (5). As a result, investigating the optimal treatment strategy for SLE patients is still an urgent problem to be solved. Here, we provide an updated review of currently available information regarding dysregulated immune cells and mechanical molecules involved in SLE pathogenesis, which would be promising for investigating new biological approaches to SLE treatment.

Mesenchymal stem cells (MSCs) are pluripotent stem cells that are widely distributed in human body, such as bone marrow (BM) (6), umbilical cord (UC) (7), umbilical cord blood (UCB) (8), peripheral blood (9), placenta (PL) (10), adipose tissues (AT) (11), and dental pulp (DP) (12). It has been demonstrated that MSCs derived EVs possess strong biological activity similar to MSCs (13). MSCs-EVs exert immunomodulatory effects by inducing immune cells differentiation into cells with more anti-inflammatory or tolerant phenotype (14). MSC-EVs promote the chemotaxis of anti-inflammatory noncoding RNAs towards injured tissues and participate in regulating inflammatory and immune response to better enhance the healing process (15). Bone marrow derived mesenchymal stem cells (BM-MSCs) are found to suppress the proliferation of cancer cells and induced dormant states (16). MSCs have self-renewal, migration and immunosuppression functions under physiological conditions. However, some MSCs are dysregulated in immune microenvironment under pathological conditions. However, tumor-derived exosomes have been demonstrated to induce phenotypic and functional changes of MSCs, which can convert into cancer-associated cells (17). MSCs senescence can be found in SLE patients or lupus mice models. Changes of MSCs morphology and microstructure occur at the early stage in SLE patients with impaired immunosuppressive effects (18). Sun et al. have found that MSCs from SLE patients exhibit structural and functional defects, including slow growth, earlier aging and decreased vitality (18). In addition, a previous study has suggested abnormalities of multiple signaling pathways are involved in regulating actin cytoskeleton and cell cycling in BM-MSCs from SLE patients, such as MAPK and BMP/TGF-β signaling pathways (19). SLE patients exist defective immune regulation of BM-MSCs from SLE patients, characteristic of down-regulated microRNA let-7f (20). The study by Che N et al. has shown that BM-MSCs from lupus-like mice and SLE patients are deficient in suppressing B cell proliferation and differentiation (21). A previous study has shown that autologous MSCs from lupus patients are not effective in treating disease. However, whether it is possible to inhibit B cell proliferation seems to distinguish between effective MSCs and ineffective MSCs (22).

MSCs have immunomodulatory effects mainly through intercellular contact and paracrine pathways. MSC-EV is the main way for them to exert paracrine effects (23). MSC-EVs have stable membrane-like structure with a phospholipid bilayer. EVs contain substantial bioactive factors, such as nucleic acids, proteins and lipids. Some EVs delivering DNAs, mRNAs, circRNAs and lncRNAs have been demonstrated to play critical roles in regulating autoimmunity. MSC-EVs transfer bioactive molecules into the recipient cells mainly through three ways, namely, endocytosis, membrane fusion and specific receptor-ligand recognition. MSC-EVs can promote the transformation of inflammatory cell phenotypes (like M1 macrophages, DCs, Th1 and Th17 cells) into immunosuppressive phenotypes (like M2 macrophages, tolerance DCs and regulatory T cells) (24, 25). Accumulating studies have implicated that EVs derived non-coding RNAs play an important role in the pathogenesis of inflammatory and autoimmune diseases (24, 25). MSC-EVs have similar biological effects to MSCs, which are promising tools for tissue engineering and regenerative medicine (15, 26). MSC-EVs delivering molecules might not only be used as diagnostic biomarkers but therapeutic targets. Up to date, little has been known about the precise role of MSC-EVs derived bioactive molecules in SLE diseases.

MSC-EVs are important carriers for material transport and information exchange between MSC and other cells (27, 28). Exosomes are the most common type of EVs. They regulate T cells-, B cells- and other immune cells-mediated immune and inflammatory reactions (29, 30). MSC exosomes (MSC-Exo) encapsulating lipids, DNAs, RNAs and proteins are essential for intercellular communications (31). The role of MSC-Exo depends on its transferring bioactive molecules, such as proteins and RNAs (32). As a nano-scale natural carrier, exosomes can encapsulate and deliver bioactive substances produced by immune cells or tissue cells, such as nucleotides, peptides and lipids (33–35). MSC-Exo shows great potentials in intercellular information change between cells. Recently, non-coding RNAs have become a hot spot in oncology, rheumatology and other fields, such as circRNAs, lncRNAs, and miRNAs (36–40). Emerging evidence has elucidated that exosome-deriving miRNAs, circRNAs and other non-coding RNAs play important roles in SLE pathogenesis (41, 42). Therefore, non-coding RNA may serve as new diagnostic biomarkers or therapeutic targets for SLE. In this review, we have summarized currently available studies on the role of MSCs and MSCs-EVs in autoimmune diseases, primarily including SLE. These findings are useful for understanding SLE pathogenesis and exploring novel biomarkers for SLE diagnosis and treatment.

## Immunoregulatory Effects of MSCs and MSC-EVs on T Cells

Immune regulation dysfunction is the main cause for SLE (43). In particular, T cells play a key role in SLE pathogenesis. It has been illustrated that the interaction between MSCs and T cells is essential for maintaining immune balance. Various soluble immune regulatory factors, growth factors, non-coding RNA, proteins are involved in the interaction process through paracrine action (44). MSCs inhibit T cell proliferation and activation through EVs in a dose-dependent manner (45, 46). Accumulated studies have shown BM-MSCs inhibit T cells proliferation and differentiation by preventing them from entering S phase and G0/G1 phase of the cell cycle (47, 48). Fetal liver-derived MSCs (FL-MSCs) inhibit CD4^+^ and CD8^+^ T cells response and promote CD4^+^CD25^+^Foxp3^+^Tregs response (**Table 1**) (49). MSCs can induce T cells differentiation from pro-inflammatory state to anti-inflammatory state mainly by inhibiting lymphocyte proliferation and pro-inflammatory cytokines production (57). Thus, the extensive wealth of in-vitro data has suggested MSCs exert immunomodulatory effect on T cells and might participate in maintaining the balance of immune microenvironment. It has been documented that MSCs reduce the activation of antigen presenting cells (APCs) by producing soluble factors, such as indoleamine-2,3-dioxygenase (IDO), prostaglandin E2 (PGE2) and IL-10. MSC promotes the proportion of regulatory T (Treg) cells and inhibits the generation of T follicular helper (Tfh) cells through an APC pathway (58). It has been well established that MSCs can induce T cells differentiation into Treg and Th2 cells but inhibit Th17 and Tfh cells differentiation and immune response. Accordingly, MSCs might serve as a useful treatment strategy for autoimmune diseases, including SLE.

**Table 1:** Mechanism of action with MSCs and EVs on T cells.

Cell type	Immune cell	Mechanism	Effect	Reference
FL-MSCs	CD4+CD8+T cell and Treg cell	–	inhibit CD4+T cells, CD8+T cells and promote Treg cells	(49)
MSC	Treg, Tfh	through an APC pathway	promotes Treg cells and inhibits the generation of Tfh	(49)
MSC-EVs	Th1 cell	regulating glycolysis and cytokine signaling pathways	inhibits T cell proliferation and Th1 differentiation	(39)
MSC-EVs	CD4^+^T cell	EVs-encapsulating miR-23a-3p and post-transcriptionally regulated TGF-β receptor 2 in T cells	suppressive Th1 differentiation	(50)
MSC-Exo	T cell	P27kip1/Cdk2 pathway	suppressive activation T cell proliferation and cell cycle arrest	(51)
MSC-EVs	T cell	increasing IL-10 and TGF-β	promote T cells apoptosis and inhibit proliferation	(13)
AD-MSC miR-10a	Th17/Treg cell	regulating Foxp3^+^ expression through TGF-β pathway	promote the differentiation of Th2 and Treg from naive CD4^+^ T cells	(52)
UC-MSCs	T cell	through the COX2/PGE2/NF-κB signaling pathway	inhibiting T cell proliferation and DC differentiation	(53)
UC-MSCs	T cell	IFNGR1/JAK2/Stats signaling pathways, IFN-γ/IDO axis	suppressive T cell proliferation and promote Treg function	(54)
AD-MSC	T cell	through regulating TGF-β and PGE2	regulate the Th17/Treg balance	(55, 56)

Tfh, T follicular helper; MSC, Mesenchymal stem cells; MSC-EVs, MSC derived Extracellular Vesicles; MSC-exo, MSC derived Exsome; FL-MSC, Fetal liver-derived Mesenchymal stem cells; BM-MSC, bone marrow Mesenchymal stem cells; UC-MSC, umbilical cord Mesenchymal stem cells; AD-MSC, adipose MSC; APC, antigen presenting cells; IL-10, interleukin 10; IDO, Indoleamine 2,3-dioxygenase; PGE2, Prostaglandin E2; TLR4, Toll-like Receptor 4; Treg, Regulatory T; Th1, T-helper 1; Th17, T-helper 1.

MSC-EVs exert immunomodulatory effects similar to MSCs. MSC-EVs inhibit T cell proliferation and Th1 differentiation by regulating glycolysis and cytokine signaling pathways (39). A previous study has suggested MSC-EVs promote CD4^+^ T lymphocytes differentiation toward a regulatory phenotype by EVs-encapsulating miR-23a-3p and post-transcriptionally regulated TGF-β receptor 2 in T cells (**Table 1**) (50). MSC-Exo suppresses the proliferation of T cells and induce cell cycle arrest through p27kip1/Cdk2 signaling pathway (**Table 1**) (51). MSC-Exo exerts immunosuppressive effects in autoimmune uveitis mice model by reducing T cell subsets and other inflammatory cells infiltration (59). Besides, MSCs-Exo enhances Treg generation *in vitro* and *vivo* (58). BM-MSC-EVs can prevent naive T cells differentiation into effector T cells and their activation (60). MSC-EVs promote the apoptosis of activated T cells, inhibit self-reactive lymphocytes proliferation, and produce more Tregs by increasing IL-10 and TGF-β (13). These findings have suggested the biological functions of MSC-EVs are similar to MSC that can regulate immune cells growth and function. Accordingly, MSC-EVs might be a promising cell-free therapy in autoimmune diseases.

**Figure 1:**
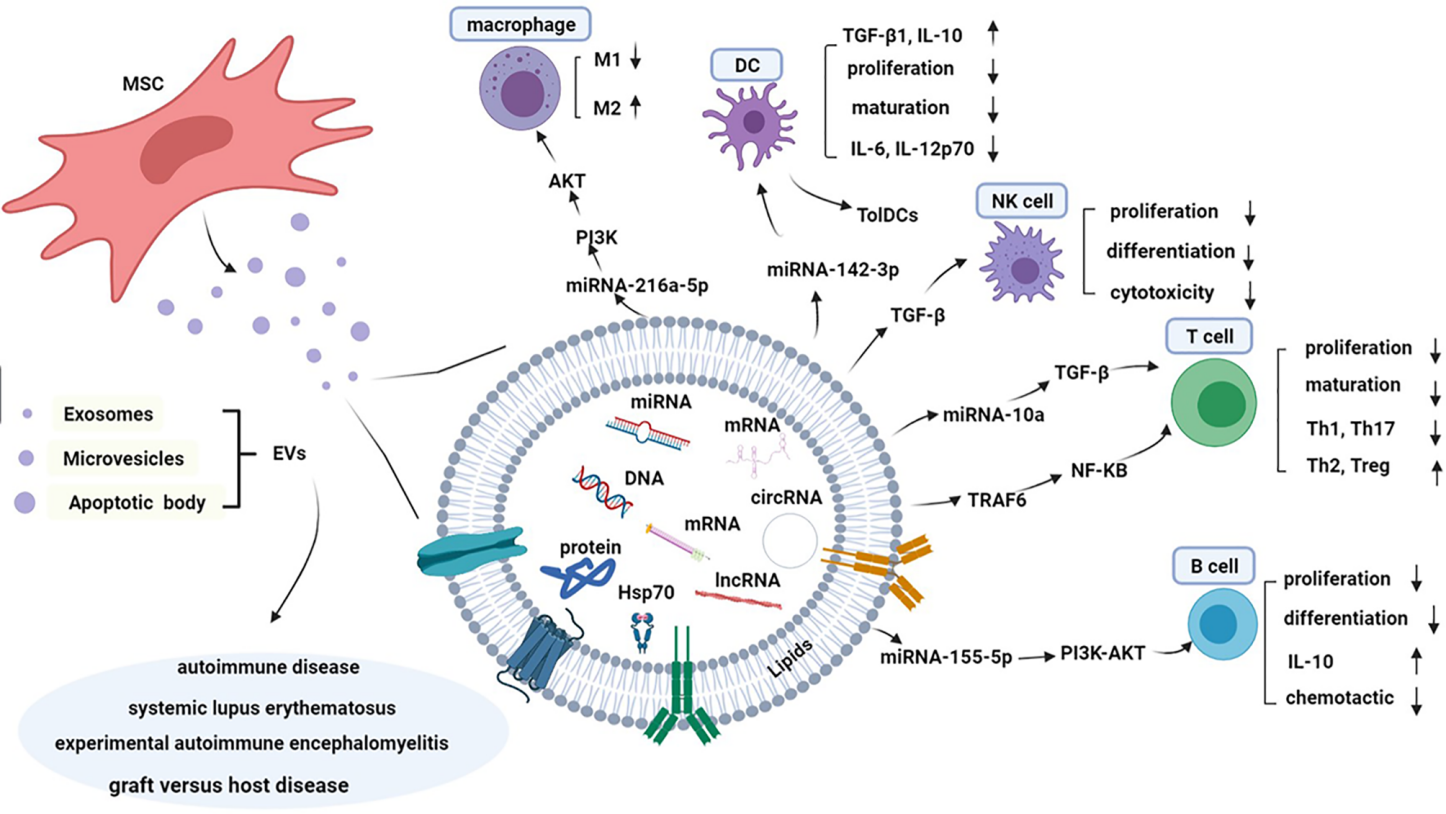
Composition and mechanism of immunological tolerance of MSC-EVs in systemic lupus erythematosus. MSC-EVs are spheroidal shaped and two-layer lipid particles containing various types of protein, lipids, DNAs, non-coding RNAs, miRNAs, and mRNA, which cause genetic information exchange by various of signal pathway and reprogramming of the recipient cell. MSC-EVs can suppress the differentiation and proliferation of B cell by PI3K-AKT pathway, and reduce production of IL-10. Similarly, T cells play the suspensive role on the proliferation and maturation, while reduce production of Th17 and Th1, and improve function of Treg and Th2 through the TGF-β/NF-κB pathway. EVs can suppress the proliferation and maturation of DCs and induce tolerable DCs with low expression of costimulatory makers. Macrophages can transform to anti-inflammatory M2 phenotype after treating by MSC-EVs through the PI3K/AKT pathway. EVs can suppress the proliferation, differentiation and cytotoxicity of NK cells in a TGF-β dependent manner. MSC-EVs play an important role in the pathogenesis of autoimmune diseases, including SLE, graft *versus* host disease, experimental autoimmune encephalomyelitis, etc.

MiRNAs are endogenous single-stranded non-coding RNAs that regulate approximately 30-70% of human genes. They play an important role in innate and adaptive immune responses by influencing cell proliferation, differentiation and apoptosis (61). Accumulated data has shown that miRNAs disruption and dysfunction can interfere with immune response, stimulate inflammatory cytokines release, initiate autoantibodies production and promote autoimmune diseases occurrence, such as SLE (62, 63). The expression of miR-146a in SLE patients serum exosomes is significantly decreased, which can promote MSCs senescence by targeting TRAF6 and inhibiting NF-κB signaling pathway activation (**Figure 1**) (64, 65). Besides, exosomal miR-146a plays a key role in regulating innate immunity by targeting toll-like receptors (TLRs), tumor necrosis factor (TNF)-associated family (TNAF), and interleukin-1 receptor-associated kinase 1 (IRAK1) (**Table 1**) (66). Accordingly, circulating exosomes encapsulating miR-146a may be a key biomarker for SLE. The study by Bolandi Z et al. has suggested adipose-derived mesenchymal stem cell exosomes (AD-MSC-Exo) miR-10a promote the differentiation of Th2 and Treg from naive CD4^+^ T cells (52). AD-MSC-Exo miR-10a can regulate Th17 and Treg differentiation by regulating Foxp3^+^ expression through TGF-β pathway (**Table 1**) (52). Taken together, MSCs carry molecular information of specific source cells and participate in intercellular communications.

Imbalance of Th1, Th2, Th17 and Treg results in inflammatory response and immune disorders in SLE (20, 67). Defective clearance of apoptotic cells (ACs) has been considered to be involved in the pathogenesis of SLE. It has been shown that human umbilical cord (UC) MSC possessed the ability to engulf ACs and then enhanced the immunosuppressive function by inhibiting T cell proliferation and DC differentiation through the COX2/PGE2/NF-κB signaling pathway (**Table 1**) (53). MSCs have significant improvements of hematological ingredient by upregulating Treg and downregulating Th17 (68). Interestingly, BM-MSCs and UC-MSCs from SLE patients have a defect in IDO activity and secretion, which is an enzyme that mediates tryptophan degradation into immunosuppression metabolites (54). However, allogeneic UC-MSC can produce extremely amount of IDO to inhibit T cell proliferation through IFNGR1/JAK2/Stats signaling pathway (**Table 1**) (54). Moreover, MSC restores the immune balance *via* regulating TGF-β and PGE2 and enhancing Treg/Th17 cells ratio in lupus mice (55, 56). In SLE patients, allogeneic UC-MSCs inhibit T cells response through IFN-γ/IDO axis (69), and induce more inducible Treg (iTreg) by generating anti-inflammatory cytokines, such as TGF-β1 (70, 71). Besides, BM-MSCs can alleviate lupus nephritis and improve mice survival rate by effectively inhibiting follicular helper T cell (Tfh) differentiation and IL-21 generation (72, 73).

## Regulation of MSCs and MSC-EVs on B Cells

Abundant activation and dysregulation of B cells is closely related to SLE pathogenesis. Pathogenic autoantibodies from B cells participate in SLE pathogenesis and mediate tissue damage, which are also involved in presenting antigens to self-reactive T cells (74). The activation of B cells leads to the production of a large number of autoreactivity autoantibodies involved in the disease process of SLE, including BAFF. A previous study has demonstrated that excessive expression of miR-152-3p is observed in SLE, resulting in increased BAFF Expression in SLE B-Cells by downregulating KLF5 (75). The study by Ma X et al. has shown that BM-MSC suppressed the excessive activation of B-cells *via* inhibiting BAFF production in MRL/lpr mice (**Table 2**) (76). Belimumab, an anti-BAFF antibody, has already been used as a treatment target for SLE, which is approved by FDA for Lupus in 2011. A published study has reported that MSCs inhibit the proliferation of B lymphocytes by keeping the cell cycle in the G1 and G0 phases and impair plasma cell formation and immunoglobulin secretion (81, 83). Besides, MSCs can indirectly inhibit B cell differentiation, maturation, and plasma cell differentiation as well as antibody production through directly contacting with T cells (**Table 2**) (77). Moreover, MSCs inhibit the proliferation, excessive activation and maturation of B cells by depleting tryptophan in the inflammatory microenvironment of human body **(** **Table 2**) (78). Most interestingly, MSCs enhance increased regulation B (Breg) cell through SDF-1-CXCR7 axis, which plays a key role in maintaining immune tolerance and inhibiting immune and inflammatory responses (84). MSCs affect B cells chemotaxis by inhibiting the production of immunoglobulin IgG, IgM and IgA and downregulating related ligands expression. As a result, MSCs regulate B cell response and mediate immune suppression, whereas the potential molecular mechanism warrants to be investigated.

**Table 2:** Mechanism of action with MSCs and EVs on B cell.

Cell type	Immune cell	Mechanism	Effect	Reference
BM-MSC	B cell	inhibition of BAFF production	suppress the excessive activation of B-cells	(76)
MSCs	B cell	directly contacting with T cells	inhibit B cell differentiation, maturation,	(77)
MSCs	B cell	by depleting tryptophan in the inflammatory microenvironment of human body	inhibits the proliferation, excessive activation and maturation of B cells	(78)
MSC	Breg cell	through SDF-1-CXCR7 axis	increased regulation B (Breg)	(77)
MSC-EVs miRNA-155-5p	B cell	targeting PI3K/AKT signaling pathway	inhibit activation of B cell	(79)
MSC	B cell	increased expression of CCL2 by CCL2-MST1-mTOR-STAT1 mediated metabolic signaling pathway	prevent inhibition differentiation, proliferation, and antibody secretion of B-cell	(80)
MSC	B cell	inhibit expression of maturation protein-1	inhibit B cells terminal differentiation	(81)
GMSCs	B cell	targeting of CD39^-^CD73 signaling pathway	suppressing B cells and produce autoantibodies	(82)

MSC, Mesenchymal stem cells; BM-MSC, bone marrow Mesenchymal stem cells; GMSC, gingiva derived MSCs; SDF-1-CXCR7, Stromal-derived factor-1 and its receptor C-X-C chemokine receptor-7; Breg, regulation B cell; CCL2, chemokine CC motif ligand 2; CCL2-MST1-mTOR-STAT1, chemokine CC motif ligand 2-mammalian Sterile 20-like kinase 1- media Time of Repair-signal transducerand activator of transcription 1.

The immunomodulatory effects of MSC on B Cells and plasma cells are associated with EVs delivering soluble factors (85, 86). MSC-exo inhibits the proliferation of T cells and B cells (**Table 2**) (29). It has been well documented that MSC-EVs delivering miRNA-155-5p reduce the activation of B cell cycle progression by targeting PI3K/AKT signaling pathway(**Figure 1** and **Table 2**) (79). Nonetheless, more studies are warranted to further investigate the potential bioactive factors in MSC-EVs and their precise effects on immune cells function in SLE and other autoimmune diseases. Understanding the mechanisms of MSC-EVs in immune regulations may help for developing new therapeutic strategies for SLE.

It has been documented that toll-like receptor 7 (TLR7) is overexpressed in SLE, which drives autoreactive B cells activation and autoantibodies production through IFN-γ signal pathway in SLE (87–89). MSC enhances IL-10 production by activating the extracellular signal-related kinase (ERK) signal but suppresses the generation of TNF-α by downregulating TLR-7/NF-κB signal in murine macrophages (90). Intrinsic B cells with autoreactive B cell receptor (BCR) expression and long-lived plasma cells continuously producing autoantibodies are essential for the development of SLE (91). A previous study has implicated that MSCs derived from SLE patients have significantly reduced expression of CCL2 (21). Those MSCs prevent from the proliferation, differentiation, and antibody secretion of B-cell through CCL2-MST1-mTOR-STAT1 mediated metabolic signaling pathway (**Table 2**) (80). Elevated expression of IL-21 in serum of SLE patients promotes B cells differentiation, which thus leads to a large number of pathogenic autoantibodies aggravating SLE manifestations (92). MSCs exert inhibitory effects on the terminal differentiation of B cells through decreasing the expression of maturation protein-1 (**Table 2**) (81). In addition, human gingiva derived MSCs (GMSCs) directly suppress autoantibodies production and proteinuria and alleviate the histopathological progression of lupus nephritis through CD39^-^CD73 signaling pathway (82). Thus, MSCs play a vital role in SLE pathogenesis by inhibiting B cells proliferation, differentiation and activation.

## Regulatory Effects of MSCs and MSC-EVs on Dendritic Cells

DCs are the most important APCs, which are key cells in defending against infection and tumor. As APCs, DCs present exogenous antigens to secondary lymphoid tissues such as spleen and lymph nodes. In lupus nephritis, DC infiltrates the kidney to exacerbate inflammation. Shahir M et al. have found that MSC-Exo induces more tolerance DCs (tol-DCs) with low expression of costimulatory markers and IL-6, but increases the anti-inflammatory cytokines of IL-10 and TGF-β (**Figure 1** and **Table 3**) (93). Moreover, the induced tol-DCs further promote the differentiation of regulatory T cells, which play a protective role in SLE (93). MSC pretreated with IFN-γ inhibits DC maturation, activation, and antigen uptakes (**Table 3**) (94). Type I IFN is closely related to the severity of lupus nephritis, hematopoietic and central nervous system symptoms of SLE patients. Plasmacytoid DCs (pDCs) are considered to be the main source of type I IFN. Apart from pDCs, T cells, B cells and NK cells are also involved in IFN production in SLE (97). However, MSCs can inhibit the generation and function of pDCs (98). Accordingly, MSCs are ideal therapeutic way for SLE patients due to their inhibitory effects on pDCs and type I IFN. The regulatory effects of MSCs on DCs make them as promising treatment strategies for SLE patients.

**Table 3:** Mechanism of action with MSCs and EVs on DC cell.

Cell type	Immune cell	Mechanism	Effect	Reference
MSC-Exo	tol-DCs	induces more tolerance DCs (tol-DCs) with low expression of costimulatory markers	increased anti-inflammatory cytokines IL-10 and TGF-β expression but decreased IL-6 expression, promotes the differentiation of regulatory T cells	(93)
MSC	DC	Combine with IFN-γ	inhibits DC maturation, activation, and antigen uptakes	(94)
MSC-Evs	DC	expression of anti-inflammatory factors (TGF-β1 and IL-10) and reduce the generation of proinflammatory cytokines (L-6 and IL-12p70)	attenuate DCs maturation and function	(95)
UC-MSCs	CD1c^+^ DCs	upregulating serum FLT3L	Increased expression of CD1c^+^ DCs	(96)

MSC, Mesenchymal stem cells; MSC-EVs, MSC derived Extracellular Vesicles; MSC-Exo, MSC derived Exsome; UC-MSC, umbilical cord Mesenchymal stem cells; IL-10, interleukin 10; TGF-β1, transforming growth factor-β1; DC, Dendritic cells; NK, Natural killer; IFN-γ, interferon; IL-6, interleukin-6.

Increasing studies have suggested the modulatory effects of MSC-EVs on immune cell functions. MSC-Exo transferring miRNA-155 and miRNA-146 have been found to regulate endotoxin-induced inflammatory response in DCs (99). Wang Y et al. have demonstrated that miR-142-3p was highly expressed in SLE, which induced pro-inflammatory moDCs involved in SLE pathogenesis (100). MSC-EVs are capable of attenuating DCs maturation and function. It has been found that some MSC-EVs derived miRNAs can decrease the expression of mature markers CD83, CD38 and CD80 and proinflammatory cytokines (IL-6 and IL-12p70) but promote the expression of anti-inflammatory factors (TGF-β1 and IL-10) (**Figure 1** and **Table 3**) (95).

A number of studies have shown that allogeneic BM-MSCs inhibit SLE inflammatory response by upregulating tolerance DCs. After transplantation of allogenic UC-MSCs, peripheral blood CD1c^+^ DCs and serum FLT3L can be significantly up-regulated in SLE patients, suggesting that UC-MSCs are useful for SLE treatment by inducing increased resistant CD1c^+^ DCs (96). Nonetheless, more future studies are warranted to elucidate the underlying mechanism involved in MSCs and DCs interactions in SLE.

## Regulatory Effects of MSCs and MSC-EVs on NK Cells

NK cells primarily mediate natural immune response and also play a crucial role in the pathogenesis of SLE (**Figure 1**). In SLE patients, the number of NK cells is significantly reduced, whose cytotoxic activity and cytokine profile are impaired. NK cells with higher expression of granzyme B are observed in active SLE patients, which can be further enhanced by IL-15 (101). Interactions between MSCs and NK cells are necessary for reducing NK cytotoxicity (102). A published study has confirmed that MSCs inhibit the activity of NK cells by regulating IDO and PGE2 (**Table 4**) (103, 104). BM-MSCs can inhibit NK cell proliferation induced by IL-12 and IL-21 but upregulate IFN-γ, IFN-α, perforin and granzyme in NK cells(**Table 4**) (105, 106). Besides, NK cells also exert regulatory effects on MSCs. NK cells promote MSCs recruitment and ROS production, which can downregulate MSCs activity (109). Interestingly, the sorted TLR4-positive MSC (TLR4^+^MSC) has a strong inhibitory effect on NK cells by regulating the receptor NKG2D (107), suggesting the pivotal immunomodulatory effects of MSCs on NK cells.

**Table 4:** Mechanism of action with MSCs and EVs on NK cell.

Cell type	Immune cell	Mechanism	Effect	Reference
MSC	NK cell	regulating indoleamine-2, 3-dioxygenase (IDO) and prostaglandin E2 (PGE2)	inhibit the activity of NK cells	(103, 104)
BM-MSCs	NK cell	inhibit IL-12 and IL-21	Suppression NK cell proliferation but increase IFN-γ and IFN-α production	(105, 106)
TLR4^+^MSC	NK cell	–	inhibitory effect on NK cells and the receptor NKG2D	(107)
FL-MSC-EXO	NK cell	regulating TGF-β	inhibit the proliferation, activation and cytotoxicity of NK cells	(108)

MSC, Mesenchymal stem cells; FL-MSC, Fetal liver-derived Mesenchymal stem cells; BM-MSC, bone marrow Mesenchymal stem cells; UC-MSC, umbilical cord Mesenchymal stem cells; IDO, Indoleamine 2,3-dioxygenase; PGE2, Prostaglandin E2; TLR4, Toll-like Receptor 4; NK, Natural killer; TLR4^+^MSC, TLR4-positive MSC; IL-12, interleukin 12; IL-21, interleukin 21; NKG2D, type II integral membrane protein; IFN-γ, interferon-γ ; IFN-α, interferon-α.

Accumulating data has implicated that MSC-EVs inhibit NK cells proliferation by regulating G0 and G1 cell cycle phases (99). Exosomes from fetal liver MSCs (FL-MSCs) inhibit the proliferation, activation and cytotoxicity of NK cells by regulating TGF-β (**Table 4**) (108). Activin A is a member of the TGF-β superfamily. It has been demonstrated that UC-MSC produces a large amount of activin A, which inhibits IFN-γ production by downregulating T-bet in NK cells (110). However, whether MSC-EVs protect against SLE by inhibiting NK cells proliferation and function warrants to be further studied in the future. It is lacking substantial evidence to support the immunomodulatory effect of MSC-EVs on NK cells in lupus, either in animal models or in clinical studies. The precise effect and underlying mechanism of MSCs/MSC-EVs on NK cells in SLE warrant to be investigated in more future studies.

## Regulatory Effects of MSCs and MSC-EVs on Macrophages

Imbalance of M1/M2 polarization and abnormal activation of macrophages are involved in the pathogenesis of SLE. Macrophages are responsible for clearing ACs. Increased ACs due to dysregulated macrophages will activate autoreactive B cells, thereby leading to abundant autoantibodies and immune complexes deposition in multiple organs and tissues (111). The polarization and activation of macrophages are different depending on certain microenvironmental conditions. It has been well established that MSCs effectively weaken the phagocytosis and antigen presentation of macrophages. Besides, MSCs can induce macrophage polarization to M2 phenotype through TGF-β/Akt/FoxO1 pathway (**Table 5**) (112). Similarly, BM-MSCs can drive macrophages differentiation into anti-inflammatory phenotype M2, while they inhibit the differentiation of pro-inflammatory phenotype M1 (120). DNA methyltransferase 1 in SLE patients’ peripheral blood mononuclear cells is increased. BM-MSC may down-regulate the expression of methyltransferase 1 through the MEK/ERK signaling pathway, thereby inhibiting self-activated peripheral blood mononuclear cells from SLE patients (121). UC-MSC can affect M1/M2 balance by regulating macrophage metabolic pathways (**Table 5**) (113). Accordingly, MSCs play a critical role in maintaining M1/M2 balance in SLE.

**Table 5:** Mechanism of action with MSCs and EVs on macrophage.

Cell type	Immune cell	Mechanism	Effect	Reference
MSC	macrophage	Through TGF-β/Akt/FoxO1 pathway	toward M2 phenotype polarization	(112)
UC-MSC	macrophage	regulating macrophage metabolic pathways	affect M1/M2 balance	(113)
MSC-Exo	macrophage	down-regulating IL-23 and IL-22	enhances the anti-inflammatory phenotype of macrophages, promoting inflammation remission	(114)
AD-MSCs	macrophage	–	toward M2 phenotype polarization	(115)
MSC-Exo	macrophage	through miR-223/pKNOX1 pathway	promoting macrophages differentiation toward M2	(116)
MSC-EVs	macrophage	through TLR4/NF-κB/PI3K/Akt signaling cascade	toward M2 phenotype polarization	(117)
UC-MSC/exosomes	macrophage	increased the proportion of M2 macrophage polarization	attenuate diffuse alveolar hemorrhage (DAH) induced inflammatory responses and alveolar hemorrhage	(118, 119)

MSC, Mesenchymal stem cells; MSC, Mesenchymal stem cells; MSC-EVs, MSC derived Extracellular Vesicles; MSC-exo, MSC derived Exsome; UC-MSC, umbilical cord Mesenchymal stem cells; AD-MSC, adipose MSC; TRAF6, IR7; IL-10, interleukin 10; IL-23, interleukin 23 ;IL-22 , interleukin 22; pKNOX1, PBX/knotted 1 homeobox 1; NF-KB, nuclear transcription factor-kappa B; PI3K, phosphoinositide 3-kinase; PGE2, Prostaglandin E2; TLR4, Toll-like Receptor 4; DAH, diffuse alveolar hemorrhage.

A number of studies have shown that MSC-Exo has lower immunogenicity and can inhibit the development and progression of experimental autoimmune encephalomyelitis, traumatic spinal cord injury and diabetes animal models by regulating M1/M2 balance and Th17/Treg ratio (122–124). A previous study has shown that MSC-EVs enhance the anti-inflammatory phenotype of regulatory macrophages by down-regulating IL-23 and IL-22 and promoting inflammation remission (**Table 5**) (114). At the same time, AD-MSCs derived EVs are capable of inducing macrophage polarization toward M2 under hypoxia conditions (**Table 5**) (115). MSC-Exo exerts anti-inflammatory properties by promoting macrophages differentiation toward M2 through miR-223/pKNOX1 pathway(**Table 5**) (116). MSC-EVs delivering miR-216a-5p can be transferred to macrophages and induce M2 macrophages polarization through TLR4/NF-κB/PI3K/Akt signaling cascade(**Figure 1** and **Table 5**) (117). Interestingly, MSC-Exo attenuates myocardial ischemia-reperfusion injury by enhancing M2 macrophages polarization through miRNA-182 and its downstream target, namely, toll-like receptor 4 (TLR4) (125). Therefore, MSCs and MSC-EVs can effectively regulate macrophages polarization toward M2 and inhibit macrophages-mediated inflammatory response. However, little is known about the effect of MSCs and MSC-EVs on macrophages in SLE. It has been shown that MSC mediated macrophage polarization depends on the IL-6 signaling pathway (126). UC-MSCs promote the proportion of CD206^+^ M2 cells and the phagocytic activity of macrophages in an IL-6 dependent manner in SLE (127). Human UC-MSCs/exosomes attenuate the diffuse alveolar hemorrhage (DAH) induced inflammatory responses and alveolar hemorrhage through increasing M2 macrophage polarization in DAH patients or lupus mice (**Table 5**) (118, 119). All these findings have strongly suggested the pivotal role of MSC-EVs in macrophage polarization in SLE. MSC-Exo serves as a nanocarrier delivering bioactive molecules between immune cells and stem cells, which may be a promising treatment way for SLE.

During the past few years, immune metabolic disorders have been implicated in SLE pathogenesis, whereas the regulatory mechanism of MSC and MSC-EVs on the immune metabolic phenotype of macrophages and M1/M2 bias in SLE remains largely unclear. Blocking the reprogramming of macrophage metabolism and maintaining the balance of M1/M2 in the immune microenvironment would provide new insight into identifying valuable strategies for the biological treatment of SLE.

## Potential Use of MSCs and MSC-EVs in SLE Treatment

Current treatment strategies for SLE are mainly aimed at controlling and mitigating disease activity. Although Belimumab and Telitacicept have been approved for SLE treatment, the heterogeneity of SLE has led to the dilemma of current treatment status (128). Accordingly, identifying a more effective treatment strategy has become a top priority for SLE. Some studies have established that MSCs can attenuate the adverse effects of immunosuppressive drugs. A previous study has implicated that MSCs combination with immunosuppressive drugs display distinct effects on T cell activation and bias (129). They exert a suppressive effect on proinflammatory T cell subsets and promote the activation and function of anti-inflammatory Treg cells (129). Besides, accumulating evidence has suggested that mycophenolate mofetil (MMF) can selectively suppress B cells proliferation (74). Lee HK et al. have found that prednisone (PD) or MMF in combination with MSCs showed better therapeutic effect than single therapy in lupus-prone MRL/lpr mice (130). Combination of MSCs with PD or MMF can prolong the survival, decrease autoantibody levels and inflammatory cytokines in serum, and reduce the inflammatory cell infiltration in kidney and spleen in lupus mice (130). Moreover, immunosuppressants can influence MSC transplantation (MSCT) mediated immune responses and prolong the efficacy of transplanted MSCs (131). Accordingly, the combination of MSCs and immunosuppressants may become a more effectively therapeutic strategy for SLE.

MSCs are characterized by high self-renewal ability, rapid expansion *in vivo* and *in vitro*, and low immunogenicity. They can participate in immune response through two ways: paracrine effects and cell-cell interaction directly. In SLE patients, autologous MSCs are defective in immunomodulatory and regenerative functions. Allogeneic mesenchymal stem cell transplantation (MSCT) has brought new hope to cure severe SLE patients (132, 133). Reconstruction of immune tolerance and tissue regeneration and repair are necessary for SLE treatment. MSCs are easy for isolation and purification, which have good therapeutic effects in MRL/lpr and (NZB/NZW) F1 mice (134). Currently available animal studies have provided strong evidence for the therapeutic potentials of MSCs in SLE. The use of MSCs in treating SLE patients has also been reported in some previously published studies. The clinical symptoms of patients are significantly improved and the disease activity index is significantly decreased, when UC-MSCs are applied to the treatment of refractory and severe SLE patients.

MSCT is effective and safe in treating SLE patients and lupus animal models, even though Deng D et al. have shown that allogeneic UC-MSCs did not exert effects on SLE patients (135). However, a previous study has revealed that metformin-treated AD-MSCs regulated the Th17/Treg balance in MRL/lpr mice, and metformin enhanced the immunomodulatory properties of AD-MSCs through AMPK and STAT1 signal (**Table 1**) (136). It has been demonstrated that transplantation of human MSC can significantly inhibit disease progression in MRL/lpr mice (137). The study by Tang X et al. has suggested that dental pulp MSC (DP-MSCs) could alleviate the disease symptoms of lupus-prone B6/lpr mice, especially reduce the kidney glomerular lesion and perivascular inflammation infiltration (138). MSCs are found to reduce proteinuria and serum anti-dsDNA levels, increase C3 and C4 levels and alter serum cytokine profiles by down-regulating the MyD88-NF-kB signaling pathway in NZBWF1 mice model (139). Most importantly, mounting clinical studies have shown intravenous infusion of UC-MSC is an available and safe practice in SLE treatment, which not only significantly declines SLE Disease Activity Index (SLEDAI) but also ameliorates renal function and systemic manifestations including hematopoietic and cutaneous systems in SLE patients (140, 141). It has been well documented that a repeated infusion of MSCs is feasible, and MSCT should be adopted again 6 months after the first time to avoid disease relapse in severe and refractory lupus patients (140, 142, 143). LN patients get to renal remission 12 months after allogeneic MSCT, including obvious renal function amelioration in parallel with significantly improved glomerular filtration rate (144). Long-term serial administration of human AD-MSCs can help to ameliorate SLE without any adverse effects, which decreases the level of anti-double-stranded DNA antibodies but significantly increases IL-10 and Treg cells (145). Taken together, MSCT is a promising treatment strategy for SLE as evidenced by both animal experiments and clinical tests. However, the safety and efficacy need to be investigated in more future studies with large sample size. Last but not the least, the effect of MSCs derived EVs including exosomes in SLE is not clear yet, although they have been demonstrated to possess similar biological effects as MSCs in SLE (146).

## Perspectives

Besides tissue repair and regeneration capacities, MSCs and MS-EVs have strong anti-inflammatory and immunomodulatory effects. They can be ideal therapeutic strategy for SLE, particularly the refractory and severe SLE patients resistant to hormones and immunosuppressant drugs. Accumulating data has suggested MSC-EVs also have anti-inflammatory, anti-apoptosis, pro-angiogenesis and immunomodulatory effects in inflammatory and autoimmune diseases by transferring bioactive constitutes to specific cells. Most importantly, MSC-EVs have much less immunogenicity but similar biological function with MSCs themselves, which act as a representative cell-free treatment way. Moreover, MSCs are demonstrated to possess tumorigenic potentials due to gene mutations, genetic instability as well as excessive proliferation and differentiation. Therefore, MSC-EVs may be a better choice for SLE treatment in future. Nevertheless, genetic modification, metabolic recombination, and other priming of MSCs *in vitro* should be considered before MSC/MSC-EVs application for SLE treatment. The standardized methods for MSC/MSC-EVs isolation, quantification and quality control should also be seriously considered before using MSC/MSC-EVs in treating SLE and other immune diseases. Lastly, the time of infusion, the appropriate dosage, the interval of treatment, and the long-term safety of MSC/MSC-EVs treatment in SLE warrant further clinical evaluations in more studies with high quality.

## Author Contributions

CY, JZ, and YT carried out literature research and reviewed all articles. JW, HL, JW, JY, and LZ helped to extract information. CY and SY wrote the paper. JS, CY, and DX revised the manuscript. SY and DX edited and submitted the article. All authors contributed to the article and approved the submitted version.

## Funding

This study is supported by funds from National Natural Science Foundation, China (82171790 and 82003042), Shandong Natural Science Foundation (ZR2020KC001 and ZR2019QH012), Science and Technology Development Program of Weifang (2020TX084), and Weifang Science and Technology Development Program (2019GX031and 2019YX020).

## Conflict of Interest

The authors declare that the research was conducted in the absence of any commercial or financial relationships that could be construed as a potential conflict of interest.

## Publisher’s Note

All claims expressed in this article are solely those of the authors and do not necessarily represent those of their affiliated organizations, or those of the publisher, the editors and the reviewers. Any product that may be evaluated in this article, or claim that may be made by its manufacturer, is not guaranteed or endorsed by the publisher.
